# Finite Element Analysis of the Effect for Different Thicknesses and Stitching Densities under the Low-Velocity Impact of Stitched Composite Laminates

**DOI:** 10.3390/polym15244628

**Published:** 2023-12-06

**Authors:** Bangxiong Liu, Jiamei Lai, Hesheng Liu, Zhichao Huang, Tianlei Liu, Yousheng Xia, Wei Zhang

**Affiliations:** 1 Polymer Processing Research Laboratory, School of Advanced Manufacturing, Nanchang University, Nanchang 330031, China; liubangxiong0798@126.com (B.L.); liusya0618@163.com (T.L.); xiay096@163.com (Y.X.); 2 School of Mechanical and Electronic Engineering, Jingdezhen University, Jingdezhen 333000, China; 3 School of Mechatronics and Vehicle Engineering, East China Jiao Tong University, Nanchang 330013, China; hzcosu@163.com (Z.H.); weizhang@email.ncu.edu.cn (W.Z.)

**Keywords:** stitching composite laminate, low-velocity impact, thickness, numerical simulation, mechanical response

## Abstract

In this study, a progressive damage model was developed for the mechanical response and damage evolution of carbon fiber stitched composite laminates under low-velocity impact (LVI). The three-dimensional Hashin and Hou failure criteria were used to identify fiber and matrix damage. The cohesive zone model was adopted to simulate the delamination damage, combined with the linear degradation discounting of the equivalent displacement method to characterize the stiffness degradation of the material, and the corresponding user material subroutine VUMAT was coded. The finite element analysis of the LVI of stitched composite laminates under different energies was finished in Abaqus/Explicit. Furthermore, the simulation predictions matched well with the results of the experimental tests. Based on this, composite laminates’ mechanical response and damage forms with different thicknesses and stitch densities were analyzed. The findings show that the main damages of composite laminates were matrix tensile damage and delamination. The stitching process could improve the impact tolerance of composite laminates, inhibiting delamination and reducing the area of the delamination damage. The higher the density of the stitching, the more noticeable its inhibition would be. The thickness of the laminate also had a more significant effect on the damage to the laminate. Thin plates were more prone to matrix tensile damage due to their lower flexural rigidity, whereas thick plates were more susceptible to delamination because of their higher flexural rigidity.

## 1. Introduction

Carbon fiber composite is extensively applied in the aviation, aerospace, and rail transportation fields due to their remarkable properties such as high strength, stiffness, lightweight, and fatigue resistance. Especially in aviation, most of the components on the newest aircraft are made of carbon fiber composites [1,2,3]. In daily maintenance and usage, these composites are susceptible to outside impacts. For example, a bird strike is one of the most typical impacts; the damage under high-velocity impact is significant and can be quickly inspected [4]. Impact events also occur, such as the dropping of tools during overhauling, and this may not be able to cause significant damage to the surface of composites. However, irreversible damage, such as matrix cracks and delamination, has already been caused inside the composites [5,6,7]. This category of damage is known as barely visible impact damage. It is more threatening under low-velocity impacts than high-velocity impacts since their damage invisibility can easily be overlooked [8,9].

To improve damage tolerance and inhibit delamination, many researchers have optimized the design of carbon fiber composites accordingly. Soubhik et al. [10] modified carbon-based nanofillers by attaching them to the surface of carbon fibers using the electrophoretic deposition technique, which was used to enhance the strength between the interfaces. Usaid et al. [11] designed a damage-resistant carbon fiber laminate and showed that the optimal cross-linking of the nanoparticles with the matrix inhibited the cracking of the matrix. Zhang et al. [12] conducted LVI experiments on eight different woven composites and conventional 2D sandwich composites, and the results showed that the energy absorption rate of 3D woven composites was 3.5–7.6% lower than that of 2D laminate composites. Wu et al. [13] investigated the damage of woven structural composites after impact using an ultrasonic nondestructive inspection technique. They revealed that the internal damage was highly dependent on the type and arrangement of the yarns, where the composites with aramid fibers as the warp yarns and carbon fibers as the weft yarns synergistically had the best performance in terms of impact tolerance. Lin et al. [14] and Wang et al. [15] introduced a negative Poisson’s ratio structure to reduce the damage, and the investigation results showed that the fiber and matrix tensile damage of negative Poisson’s ratio structured laminates could be reduced by about 40% under LVI. Moreover, processes such as stitching [16,17,18,19,20,21,22] and Z-pin [23,24,25] could also effectively improve the delamination resistance of composites. Compared with other structural optimization methods, the stitching process is a simple, convenient, and effective process to improve the interlaminar performance of composites.

Numerous researchers have conducted a series of experiments to investigate the mechanical response and damage mechanisms of composite laminates stitched after experiencing impact. The impact resistance of stitched composite laminates is affected by the thickness of the laminate, the impact energy, the direction of layup, and factors related to the stitching, such as the stitching type, density, diameter, strength, pattern, etc. Kim et al. [26] adopted the I-fiber process [27] to produce composites with different stitch densities. The results of the impact tests demonstrated a significant reduction in the damage area. Francesconi et al. [28] revealed that the stitching hindered the delamination extension of stitched composite laminates, and the fiber breakage would extend along the path of the stitches, which reduced the delamination damage, but the flexural strength would be reduced. Erdogan et al. [29] and Bilisik et al. [30,31] applied multiple stitches in different directions to limit the damage of delamination and fiber breakage. He et al. [32] explored the effect of stitching ways and patterns on the impact performance of stitched composites under a broad range of impact energies. Inserting stitches could increase the peak load of the impact process, and the impact resistance was improved. The narrower the stitching density was, the better the impact performance was. Tan et al. [33] investigated stitch density and diameter effects on the low-velocity impact performance of stitched composite laminates. The investigation findings showed that the stitch diameter had little effect on the peak impact force, and the higher the stitch density and diameter, the more effective it was in bridging delamination cracks and inhibiting delamination expansion. Most of these investigation works on the LVI of stitched laminates have focused on experimental studies, while there needs to be more literature on simulation prediction. Considering the high cost of experimental testing and the inability to monitor invisible damage accurately, numerical simulation has been recognized as an effective method for predicting complex damage mechanisms within stitched composites [34,35,36,37,38,39].

Aymerich et al. [34] investigated the effect of stitching on a nodal fracture of lap-jointed composites. The effect of stitched yarns on the strain energy release rate was evaluated. Yoshimura et al. [35] used beam elements to simulate stitches by adopting two-node beam elements arranged uniformly in the *x* and *y* directions. The cross-section of the beam elements was a circle with a diameter of 0.6 mm. Tan et al. [36,37] employed a three-node rod element to simulate stitches. They identified a four-step process of interfacial spalling, relaxation absorption, fiber breakage, and pull-out for stitch breakage. A 2D FEM modeled the laminates, and while this reduced the calculation time, it only obtained the damage on the top and bottom surfaces of the ply and not the entire ply with thickness. Francesconi et al. [38] considered the stitching as a solid element inserted into the laminate, which was modeled by using a C3D8R element, and cohesive elements were inserted between the stitching and the laminate to simulate the bonding relationship between the stitching and the laminate. Mao et al. [39] proposed a FEM model to simulate the evolution of stitched laminates under low-velocity impact. A strain-based 3D Hashin criterion was used as a damage criterion, and a predefined constant degradation criterion was used to characterize the intralayer damage evolution. However, this approach can only address specific situations and needs to be more generalizable.

Since the 2D FEM did not consider the plywood thickness effect, the predefined constant degradation criteria had limitations. In this work, we modeled the stitched resin columns according to the lock stitch characteristics, combined the damage criterion of 3D Hashin and Hou failure criterion, and the linear degradation scheme based on the equivalent displacement method as the damage evolution model. The cohesive zone elements with zero-thickness were selected, and the delamination was predicted using the B-K criterion. The above damage models were coded into the user-defined subroutine VUMAT. Then, a three-dimensional finite element model was established in Abaqus/Explicit to analyze the mechanical response and damage of the stitched carbon fiber composite laminates, and the accuracy of the model was verified through experiments. Ultimately, the mechanical response and damage of composite laminates with different stitch densities and thicknesses were discussed and analyzed in detail.

## 2. Model of Progressive Damage to Composite Laminates

In general, the damage of composite laminates can be divided into two categories: intralaminar damage and interlaminar damage. Intralaminar damage includes tensile and compressive fracture of fibers and tensile and compressive crushing of the matrix; interlaminar damage is the damage that occurs between two adjacent layers, which is often shown as delamination damage. The damage process can be described by a progressive damage model, which includes a continuous damage model, a damage initiation criterion, and a damage evolution model.

### 2.1. Continuous Damage Model

Composite structures were more complex, and their damage accumulated until they were destroyed entirely. Therefore, simple damage criteria cannot be used to predict the integrity of the structure. The continuous damage model was used to evaluate the damage mode of this kind of damage accumulation. The stiffness degradation of the composite during loading was modeled by introducing the damage variable di into the constitutive relationship of the composite. The modified constitutive equations were used to describe its stress-strain relationship [40], and the flexible degradation matrix *S_d_* containing the damage variables under LVI is shown in Equation (1).

(1) $$S_{d}=\left[\begin{array}{cccccc} \frac{1}{d_{f}E_{11}} & -\frac{\nu_{21}}{E_{22}} & -\frac{\nu_{31}}{E_{33}} & & & \\ -\frac{\nu_{12}}{E_{11}} & \frac{1}{d_{m}E_{22}} & -\frac{\nu_{32}}{E_{33}} & & & \\ -\frac{\nu_{13}}{E_{11}} & -\frac{\nu_{23}}{E_{22}} & -\frac{1}{E_{33}} & & & \\ & & & \frac{1}{d_{f}d_{m}G_{12}} & & \\ & & & & \frac{1}{d_{f}d_{m}G_{23}} & \\ & & & & & \frac{1}{d_{f}d_{m}G_{31}} \end{array}\right]$$

where *d_i_* (*i* = *m*, *f*) are the damage variables of the fiber and matrix, respectively; *E_ij_*, *v_ij_*, *G_ij_* (*i*, *j* = 1, 2, 3) represent the modulus of elasticity, Poisson’s ratio, and shear modulus in the longitudinal, and out-of-plane directions, respectively.

The stiffness degradation matrix *C_d_* was derived from the inverse of the flexibility matrix *S_d_*, as shown in Equation (2),

(2) $$C_{d}=\frac{1}{D}\left[\begin{array}{cccccc} d_{f}E_{11}\left(1-d_{m}\nu_{23}\nu_{32}\right) & d_{f}d_{m}E_{11}\left(\nu_{21}+\nu_{23}\nu_{31}\right) & d_{f}E_{11}\left(\nu_{31}+d_{m}\nu_{21}\nu_{32}\right) & & & \\ & d_{m}E_{22}\left(1-d_{f}\nu_{13}\nu_{31}\right) & d_{m}E_{22}\left(\nu_{32}+d_{f}\nu_{12}\nu_{31}\right) & & & \\ & & E_{33}\left(1-d_{f}d_{m}\nu_{12}\nu_{21}\right) & & & \\ & & & Dd_{f}d_{m}G_{12} & & \\ & & & & Dd_{f}d_{m}G_{23} & \\ & & & & & Dd_{f}d_{m}G_{13} \end{array}\right]$$

(3) $$\left\{\begin{array}{c} d_{f}=\left(1-d_{ft}\right)\left(1-d_{fc}\right)\\ d_{m}=\left(1-d_{mt}\right)\left(1-d_{mc}\right)\\ D=1-d_{f}d_{m}\nu_{12}\nu_{21}-d_{m}\nu_{23}\nu_{32}-d_{f}\nu_{13}\nu_{31}-2d_{f}d_{m}\nu_{21}\nu_{32}\nu_{13} \end{array}\right.$$

where *d_ft_*, *d_fc_*, *d_mt_*, *d_mc_* represent fiber tensile damage variable; fiber compression damage variable; matrix tensile damage variable; and matrix compression damage variable, respectively.

As the stiffness degradation matrix *C_d_* contained damage variables, in order to solve the convergence difficulties due to the softening behavior of the damage layer, the viscous regularization method [41,42] was adopted, which enabled the tangent stiffness matrix of the softened material to be positive at a sufficiently small time increment. The stiffness degradation process was smooth and efficiently converged.

### 2.2. Failure Criterion

The 3D Hashin failure criterion [43] is adopted as the starting criterion for fiber damage, and the failure criterion is as follows:

Fiber tensile damage (*σ*_11_ ≥ 0)

(4) $$F_{ft}={\left(\frac{\sigma_{11}}{X_{T}}\right)}^{2}+{\left(\frac{\sigma_{12}}{S_{12}}\right)}^{2}+{\left(\frac{\sigma_{13}}{S_{13}}\right)}^{2}\geq 1$$

Fiber compression damage (*σ*_11_ < 0)

(5) $$F_{fc}={\left(\frac{\left|\sigma_{11}\right|}{X_{C}}\right)}^{2}\geq 1$$

The Hou failure criterion [44,45] is used as the starting criterion for matrix damage, and the failure criterion is as follows:

Matrix tensile damage (*σ*_22_ ≥ 0)

(6) $$F_{mt}={\left(\frac{\sigma_{22}}{Y_{T}}\right)}^{2}+{\left(\frac{\sigma_{12}}{S_{12}}\right)}^{2}+{\left(\frac{\sigma_{23}}{S_{23}}\right)}^{2}\geq 1$$

Matrix compression damage (*σ*_22_ < 0)

(7) $$F_{mc}=\frac{1}{4}{\left(\frac{-\sigma_{22}}{S_{12}}\right)}^{2}+\frac{Y_{C}\sigma_{22}}{4S_{12}^{2}}-\frac{\sigma_{22}}{Y_{C}}+{\left(\frac{\sigma_{12}}{S_{12}}\right)}^{2}\geq 1$$

where *σ_ij_* (*i, j* = 1, 2, 3) are the stress in each direction; *X*_T_ and *X*_C_ denote the longitudinal tensile and compressive strengths; *Y*_T_ and *Y_C_* represent the transverse tensile and compressive strengths; and *S*_12_, *S*_13_, and *S*_23_ represent the longitudinal and transverse shear strengths.

### 2.3. Damage Evolution Model

**Figure 1:**
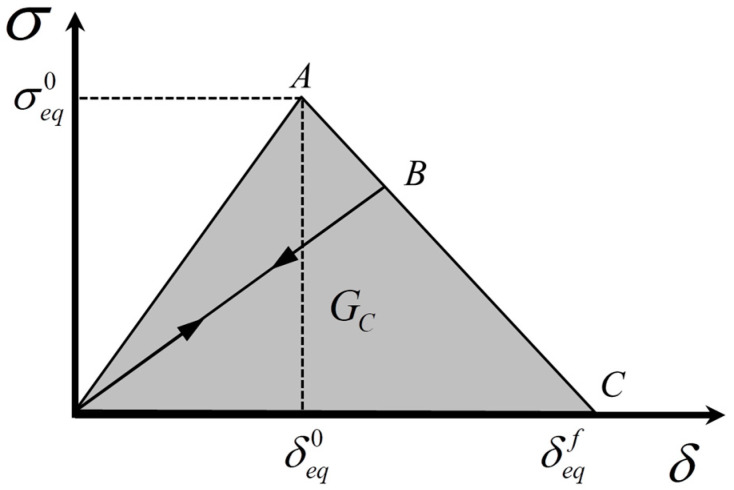
The equivalent stress-displacement relationship for intralaminar damage.

Once damage occurs, continued loading would reduce the stiffness, and the capacity of the structure to bear the damage would be reduced and a proper damage evolution model needs to be defined to simulate the process of damage accumulation in the damaged region. The damage of composite laminates under low-velocity impact exhibited localization, and the size of the meshes affected the numerical results. In order to reduce the mesh dependence in the material softening process, the characteristic length of the element was introduced into the damage evolution model, and the constitutive relationship was transformed into a stress-displacement relationship [46,47], as shown in Figure 1. Intralaminar damage was divided into fiber tensile and compression, matrix tensile and compression damage, and this diagram could represent each of these damage failure modes. The positive slope of the stress-displacement before damage initiation represented that the material was in the linear elasticity phase, and damage started to occur in the material when it reached point A. The negative slope after damage initiation indicated that the damage began to evolve, and point C was reached, indicating complete failure of the material. The energy consumed by each element was expressed in Equation (9).

(8) $$\delta_{eq}^{f}=l_{C}\varepsilon_{eq}^{f}$$

(9) $$G_{C}=\frac{1}{2}l_{C}\varepsilon_{eq}^{f}\sigma_{eq}^{f}$$

where *G_C_* is the fracture energy of the material; $\delta_{eq}^{f}$ is the displacement at failure; $\varepsilon_{eq}^{f}$ denotes the equivalent strain at failure; $\sigma_{eq}^{f}$ denotes the equivalent stress at failure; and *l_C_* represents the characteristic length of the element. The detailed derivation of the damage evolution derivation process for fiber and matrix was shown in the literature [40].

### 2.4. Interlaminar Damage

Interfacial debonding, also known as interlaminar damage, significantly affects fiber-reinforced composites due to the significant difference in stiffness between the fibers and the resin matrix. In this study, a cohesive element based on the bilinear traction–separation criterion was introduced in the interlaminar layers [48,49], and the quadratic stress criterion was adopted for initiating the delamination damage, as shown in Equation (10),

(10) $${\left(\frac{\langle t_{n}\rangle }{N}\right)}^{2}+{\left(\frac{t_{s}}{S}\right)}^{2}+{\left(\frac{t_{t}}{T}\right)}^{2}=1$$

where *t_n_* denotes the normal stress; *t_s_* denotes the first tangential stress; *t_t_* denotes the second tangential stress; *N* denotes the normal strength; and *S*, *T* denote the tangential strength.

When the initiation criterion was satisfied, the composite laminate started delaminating. The B-K fracture energy criterion [50] was adopted to calculate the energy dissipation under mixed-mode loading, as shown in Equation (11),

(11) $$G^{C}=G_{n}^{C}+\left(G_{s}^{C}-G_{n}^{C}\right){\left(\frac{G_{S}}{G_{T}}\right)}^{\eta }$$

where $G_{}^{C}$, $G_{n}^{C}$, $G_{s}^{C}$ denote the total, normal, and shear fracture energy, respectively; *G_S_* denotes the shear dissipation energy, *G_T_* denotes the total dissipation energy; and *η* denotes the curtain index in the B-K criterion, which is taken as 1.45 [51].

### 2.5. Stitching Resin Cylinder Model

**Figure 2:**
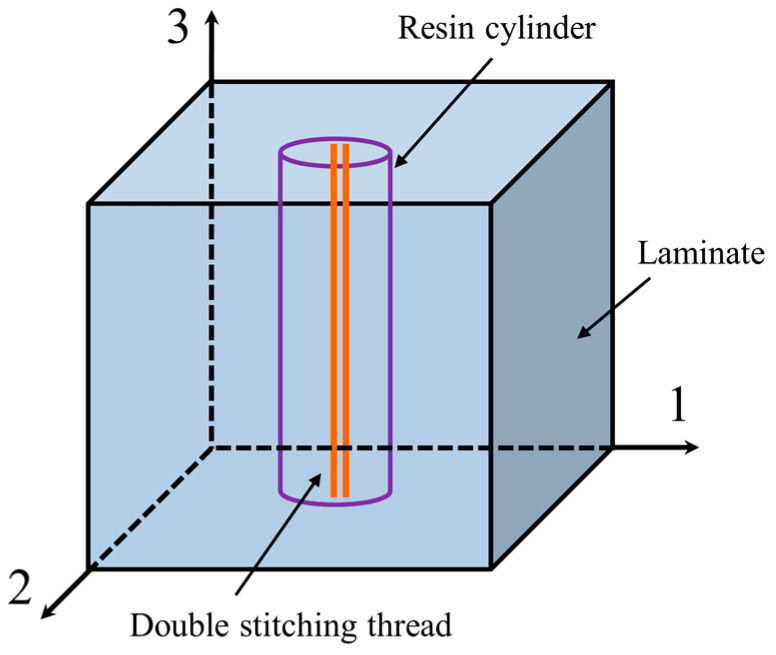
Stitching resin cylinder model.

The stitches were inserted into the prefabricated parts using a modified lock stitching process, in which two stitching threads were retained in the prefabricated parts, depending on the characteristics of the process. After adding the suture, the resin will surround the thread, forming a stitch resin cylinder after curing. Stitched carbon fiber composites could consist of carbon fiber composite laminates and stitched resin cylinders. As shown in Figure 2, the stitched resin cylinders were regularly disposed of in the laminate according to the practical requirements. Direction 1 was the stitching pitch direction, direction 2 was the stitching space direction, and direction 3 was the thickness direction. The homogenization theory [52] was adopted.

(12) $$\left\{\begin{array}{c} E_{eq}=E_{S}V_{S}+E_{M}V_{M}\\ X_{eq}=X_{S}V_{S}+X_{M}V_{M}\\ \nu_{eq}=\nu_{S}V_{S}+\nu_{M}V_{M}\\ \rho_{eq}=\rho_{S}V_{S}+\rho_{M}V_{M} \end{array}\right.$$

where *E* represents elastic modulus; *X* represents tensile strength; *v* represents Poisson’s ratio; *ρ* represents density; subscript *eq* denotes an equivalent stitching resin cylinder; subscripts *S* and *R* denote the stitching and resin curing agent mixtures, respectively.

The constitutive relationship of the stitching resin column is shown in Equation (13),

(13) $$\left\{\begin{array}{c} \sigma_{11}=E\varepsilon_{11},\varepsilon_{11}\geq 0\\ \sigma_{11}=0,\varepsilon_{11}<0 \end{array}\right.$$

where *σ*_11_ denotes the tensile stress; *E* denotes the elastic modulus; and *ε*_11_ denotes the tensile strain.

The failure criterion of the stitching resin column is shown in Equation (14),

(14) $$D_{\mathrm{st}}={\left(\frac{\varepsilon_{11}}{\varepsilon_{\max}}\right)}^{2}\geq 1$$

where *ε*_11_ denotes the strain undergone by the stitched resin cylinder and *ε_max_* denotes the maximum permissible strain.

## 3. Finite Element Modeling of LVI

Before establishing the finite element model, some fundamental assumptions must be made according to the experimental process of impact with a falling impactor: (1) ignore the deformation of the impactor as a rigid body; (2) ignore the slight friction between the impactor and the laminate; (3) ignore the damage produced by the stitching process, and the material properties of the stitched laminate are the same as those of the unstitched laminate.

**Figure 3:**
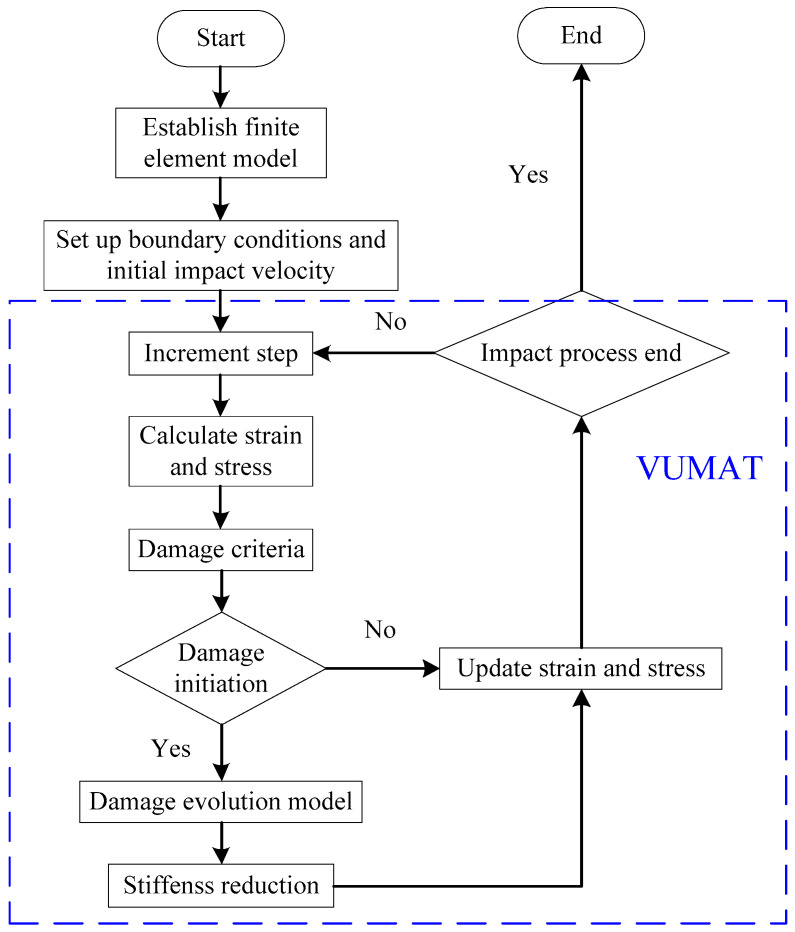
Flow chart of numerical simulation for stitched carbon fiber composite laminates.

A finite element model of LVI was established in Abaqus/Explicit 2016. The VUMAT subroutine described the 3D Hashin, Hou failure criteria, and damage evolution model. The flow chart of the numerical simulation is shown in Figure 3. Firstly, the finite element model of the stitched carbon fiber composite laminate was established. Then, the boundary conditions were set up for the laminate, and the initial velocity of the impactor was calculated based on the energy and mass of the impactor. The solver calculated the current strain increment at each time increment step and passed it to VUMAT to compute the stresses and strains for the element integration. The stiffness degradation using the damage evolution model was started after the damage initiation criterion was satisfied. Finally, the stresses and strains were updated and saved for the next incremental step to be used until finished.

### 3.1. Geometric Dimensions and Boundary Conditions

**Figure 4:**
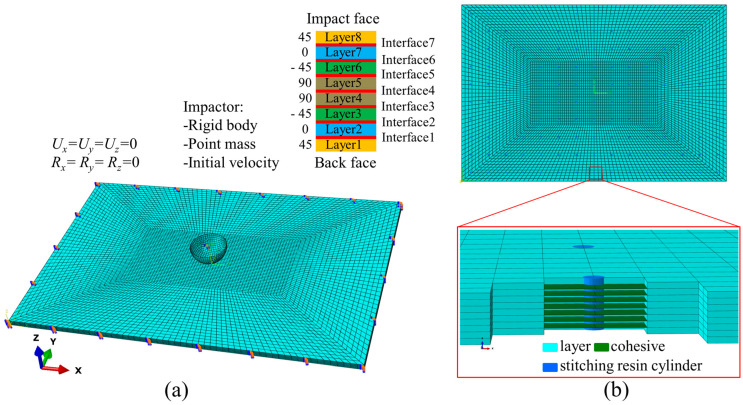
Finite element models of LVI of stitched carbon fiber composite laminate. (**a**) Setting up the boundary conditions and initial velocity of the impactor; (**b**) embedding the stitching resin cylinder into the laminate.

The finite element model consisting of the stitched carbon fiber composite laminate and impactor was established, as shown in Figure 4. The geometric size of the specimen was 150 mm × 100 mm. To investigate the effects of different thicknesses and stitch densities on the stitched laminates, different thicknesses of 2.4 mm and 4.8 mm were selected. The corresponding layup sequence was [45/0/–45/90]_s_ and [45/0/–45/90]_2s_, with each layer having a thickness of 0.3 mm. The stitches were Kevlar 29, 1500 denier. For convenience of expression, the 2.4 mm unstitched and stitched laminates were denoted as unstitched laminate (UL), stitched laminate with stitch pitch 10 mm and stitch space 10 mm (SL1010), stitched laminate with stitch pitch 10 mm and stitch space 15 mm (SL1015), and stitched laminate with stitch pitch 15 mm and stitch space 15 mm (SL1515), respectively. Laminates with a thickness of 4.8 mm were recorded with the symbol T after the abbreviation UL-T, SL1010-T, SL1015-T, and SL1515-T, respectively. The layup direction of the composite laminate was defined by adopting a local coordinate system, with direction x as the fiber direction, direction y as the perpendicular direction to the fiber, and direction z as the thickness direction. The impactor was a hemispherical cylinder, and only the bottom of the impactor was in contact with the composite laminate, so the impactor was simplified to a hemispherical invariable rigid body with a diameter of 16 mm and a mass of 5.3 kg in the simulations and the initial velocity could be calculated according to the formula *E* = 1/2 *mv*^2^, which was given to the impactor. The impactor was located on the center of the laminate, and the distance between the bottom of the impactor and the upper surface of the laminate was 0.1 mm to ensure that the impactor was always perpendicular to the laminate during its movement. The boundary conditions were consistent with the experiment, and fixed restraints were used.

### 3.2. Element Types Selection and Gridding

An eight-node solid element C3D8R was used for the laminate, and in order to ensure the accuracy of the analysis and save the cost of computation, the mesh of the impact region was refined, and other regions were coarsened. The refined area was 72 mm × 36 mm with a mesh size of 1.2 mm × 0.9 mm × 0.3 mm. The hourglass control method with relaxed stiffness was selected to avoid mesh distortion. An eight-node cohesion element COH3D8 with zero-thickness was inserted between the adjacent layers to simulate interlaminar damage. The stitches were simulated using T3D2 rod elements, and all stitches were embedded in the laminate.

### 3.3. Contact Settings and Material Properties

The impactor did not penetrate the laminate during the low-velocity impact process. Thus, the generalized contact algorithm was adopted for contact between the impactor and laminate. The normal contact was set to be a hard contact, and the friction coefficient of tangential contact was 0.3 [41]. The material parameters of the laminate are shown in Table 1, including the material parameters of the interface and the unidirectional lamina. The material parameters of the stitching resin cylinder are shown in Table 2, which consists of Kevlar 29 and an epoxy resin curing agent mixture (R688-H3268), with Kevkar29 accounting for 70% of the volume fraction, and substituting it into Equation (12) calculates the equivalent parameters.

## 4. Results and Discussion of Numerical Simulation

### 4.1. Validation of the Model

**Figure 5:**
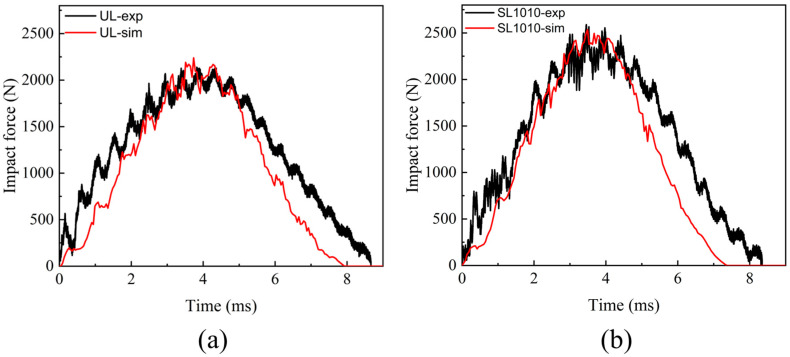
Impact force-time curves for experiments and simulations under 10 J energy. (**a**) Comparison of experiments and simulations on unstitched laminates; (**b**) comparison of experiments and simulations on stitched laminates.

The stitched and unstitched composite laminates were produced using the VARTM molding process. The low-velocity impact experiments were carried out on a drop-weight impact machine (Instron CEAST 9340, Instron Corporation, Norwood, MA, USA) according to the standard ASTM D7136 [53] for testing the damage resistance of composites, and the results such as impact force, impact time, and impact displacement were obtained. To confirm the accuracy of the model, the results were compared between the experimental and simulation results. The comparison results showed that the numerical model predictions matched well with the experimental results. Figure 5 represents the impact force-time curves of numerical simulation and experimental measurement under the impact energy of 10 J. As can be seen from the graph, the beginning of the impact process showed an oscillating upward trend, which was mainly due to the elastic vibration caused by the first contact between the surface of the laminate and the bottom of the impactor. The amplitude in the early stage was relatively stable, indicating that the damage was accumulating gradually inside the material. As the impact force approached the peak, intralaminar and delamination damage had already occurred in the laminates, resulting in a sharp shock near the peak. And the increasing trend gradually slowed down because the overall stiffness of the composite material continued to decay throughout the impact process. After the impact force increased to the peak load, the impactor rebounded, and the impact force decreased gradually to 0 J. At that time, the impactor ultimately left the composite laminate surface. This mechanical response was different from the edge-on impact in our previous investigation [22], in which the mechanical response curve in the edge-on impact has an additional phase of an oscillatory plateau than that of the central impact. In comparison, this phase in the mechanical response of the central impact showed a peak load. Compared with Figure 5a,b, the peak value of the stitched composite laminate was higher than the unstitched. It was mainly caused by the stitches embedded in the laminate, forming a stitched resin cylinder that could resist a part of the impact force.

**Figure 6:**
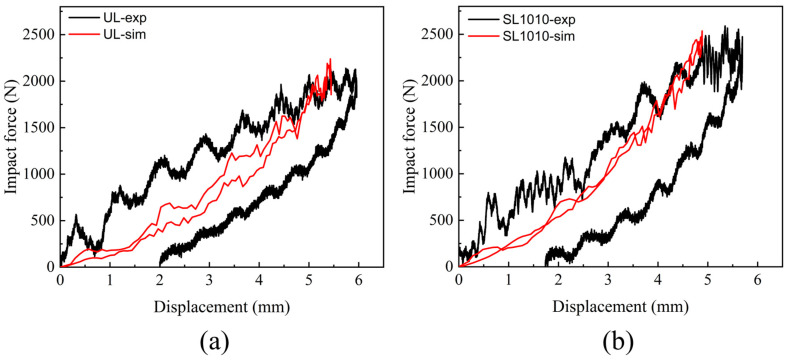
Impact force-displacement curves for experiments and simulations under 10 J energy. (**a**) Comparison of experiments and simulations on unstitched laminates; (**b**) comparison of experiments and simulations on stitched laminates.

The impact force-displacement curves for numerical simulations and experimental measurements under energy of 10 J are shown in Figure 6. The maximum displacement predicted by the numerical simulation was slightly less than the experimentally measured displacement. When the impact velocity went to 0, the impactor reached the maximum center displacement, the value of which was much larger than the thickness of the composite laminate. It was primarily due to the impact on the laminate, which caused bending deformation and a subsequent reduction in displacement when the impactor rebounded. In comparison with Figure 6a,b, the residual displacements of the stitched composites in the experiments were smaller than those of the unstitched composites, indicating that the stitching process was beneficial in reducing the out-of-plane distortion of the composite laminates. Furthermore, when the impact force was 0, it indicated that the impactor had left entirely the material surface, while residual displacement could still be seen in the experimental results. When the impactor left the surface of the composite laminate, the laminate was still bent and could not be completely restored to a stable state. It may be that the composite laminates in the simulation showed a more robust elastic behavior compared to the experiments, enabling the laminates to be fully restored to their original state. During the tests, the specimens would have permanent indentations due to the plastic behavior in the matrix under the presence of fiber entanglement and frictional resistance between layers. These behaviors were not considered in the simulation process. Therefore, the specimens in the simulation will return to their initial state after unloading.

### 4.2. Effect of Different Stitch Densities on Mechanical Response and Damage of Composite Laminates

#### 4.2.1. Mechanical Response

**Figure 7:**
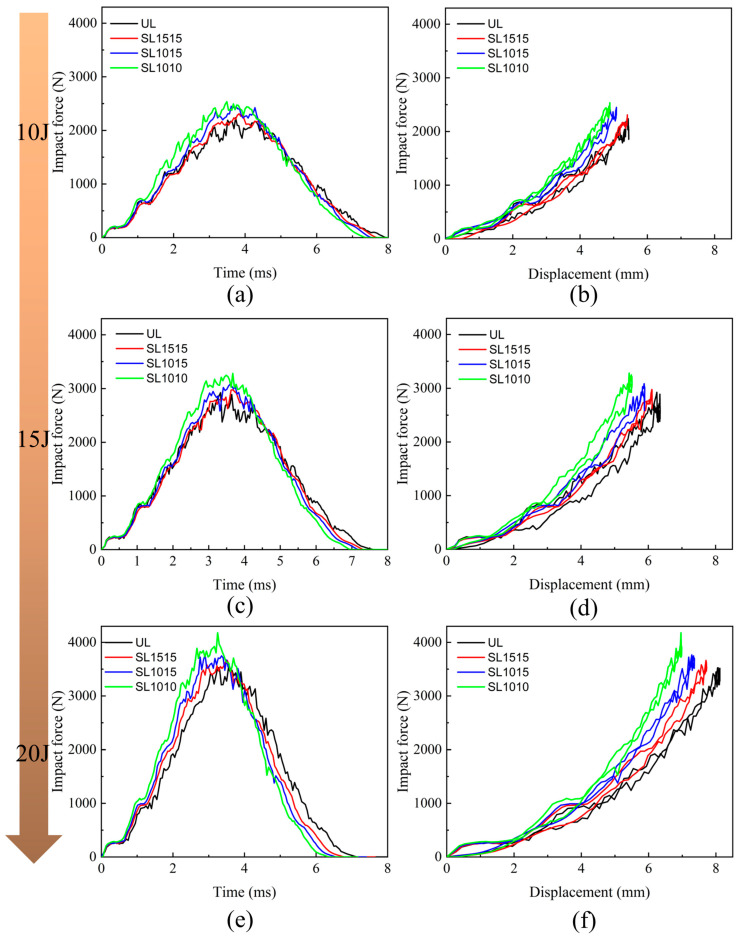
Mechanical response to the out-of-plane central impact of stitched and unstitched carbon fiber composite laminates. (**a**) Impact force-time curves under the energy of 10 J; (**b**) impact force-displacement curves under the energy of 10 J; (**c**) impact force-time curves under the energy of 15 J; (**d**) impact force-displacement curves under the energy of 15 J; (**e**) impact force-time curves under the energy of 20 J; (**f**) impact force-displacement curves under the energy of 20 J.

The mechanical response of LVI is presented in Figure 7, which represented the impact force-time curves and impact force-displacement curves under different energies for the groups UL, SL1515, SL1015, and SL1010, respectively. As the graphs show, the stitched and unstitched laminates exhibit similar characteristics, with the peak value of the impact force increasing as the energy increases. Under the same impact energy, the peak force of the stitched laminate was more significant than the unstitched one. It increased with the increase in stitching density, which indicated that embedding stitching was beneficial to enhance the impact tolerance of the laminate. The laminate with the tightest stitch density exhibited the highest impact resistance. That was because the higher the stitch density, the more stitching resin cylinders there were in the central area, which could work together to resist the impact force. The impact force-displacement curves for stitched and unstitched laminates under different impact energies are given in Figure 7b,d,f. As the energy was increased, the value of the maximum displacement of the impactor was also increased, and the damage was also increased. The maximum displacement value was the largest under 20 J, and the bending deformation of the laminate was also the largest among the three groups of specimens.

#### 4.2.2. Intralaminar Damage

**Figure 8:**
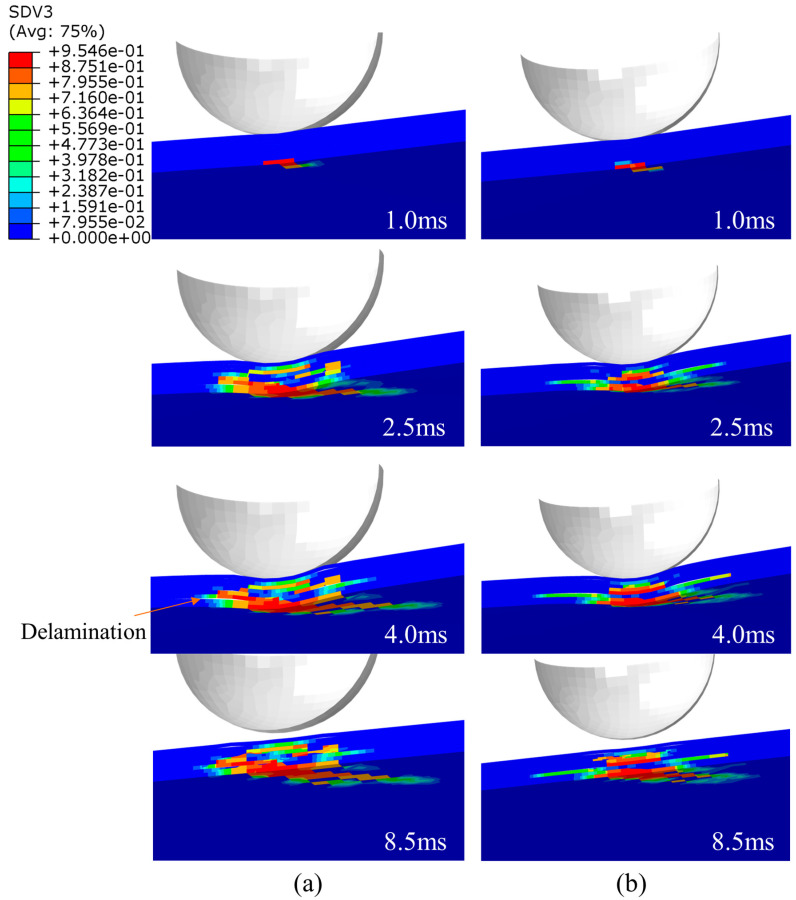
Matrix tensile damage of stitched and unstitched composite laminates at different times under 10 J. (**a**) Matrix tensile damage at different times in group UL; (**b**) matrix tensile damage at different times in group SL1010.

**Figure 9:**
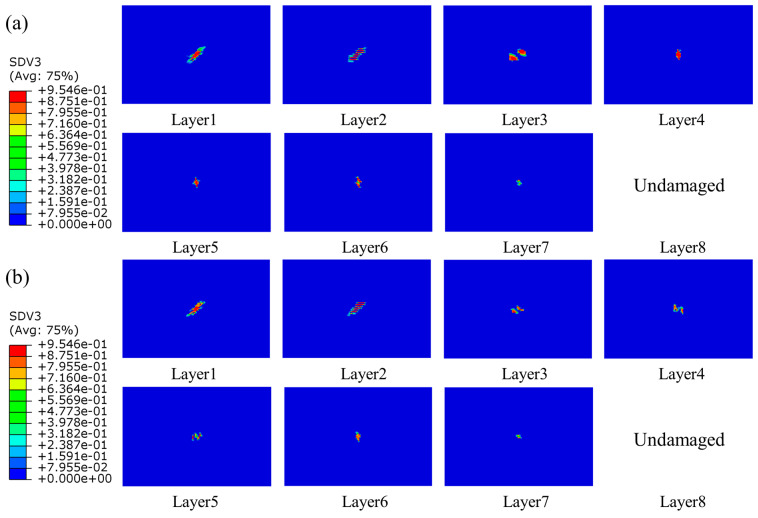
Matrix tensile damage of stitched and unstitched composite laminates under 10 J. (**a**) Matrix tensile damage in group UL; (**b**) matrix tensile damage in group SL1010.

**Figure 10:**
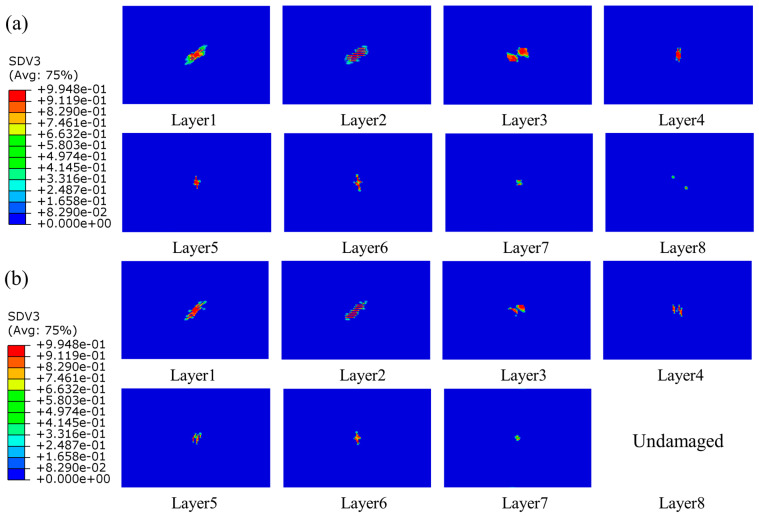
Matrix tensile damage of stitched and unstitched composite laminates under 15 J. (**a**) Matrix tensile damage in group UL; (**b**) matrix tensile damage in group SL1010.

**Figure 11:**
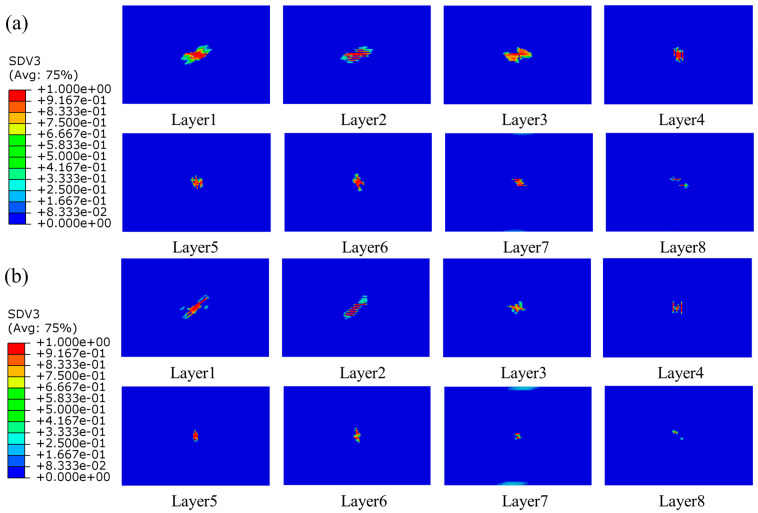
Matrix tensile damage of stitched and unstitched composite laminates under 20 J. (**a**) Matrix tensile damage in group UL; (**b**) matrix tensile damage in group SL1010.

Since the impact energy in this work was not high, the fiber damage did not occur in the simulation results. The primary damage was matrix tensile damage, and there was minor matrix compression damage at higher energies. It was also quite different from the damage caused by edge impacts, which caused much more damage to the laminates, with the damage forming areas of crushing, expanding, and protruding outwards on both sides of the plane. Intralaminar damage was mainly compressive [22]. The damage in the laminate of the group SL1010 and UL at different times under 10 J was shown in Figure 8. At 1.0 ms, the damage area of the laminate occurred in the bottom layer, the damage would expand with the impact process, the laminate was subjected to tensile stress, and delamination damage gradually began to occur. When the impact time was 4.0 ms, the damage was the most serious, and the delamination reached the maximum level. After 4.0 ms, the impact process was the rebound stage of the impactor, and the area of the damage remained unchanged in this period. In addition, the delamination damage of stitched laminates was minor compared to that of unstitched laminates. The distribution of matrix tensile damage for group SL1010 and UL under three impact energies of 10 J, 15 J, and 20 J are shown in Figure 9, Figure 10 and Figure 11, respectively. The red color part indicates that the material had been damaged, and the blue part indicates that no damage had been produced. Layer 1 represented the bottom layer of the laminate. Layer 8 represented the top layer of the laminate, which was the panel in contact with the impactor. The central position of the laminate produced a displacement along the impact direction during the whole impact process, which was mainly due to the tensile stress on the laminate, leading to matrix tensile damage. It could be seen from the figure that the sewn and unsewn laminates showed similar behavior at different impact energies, and the extent of damage increased with the increase in impact energy. As can be seen, there was no damage in Layer 8 of the sutured laminate, while damage was seen in Layer 8 of the unstitched laminate from Figure 10, indicating that the stitches were able to absorb some impact energy, thus reducing the impact energy on the matrix. In addition, each of the remaining layers showed tensile damage, with the three layers close to the bottom, Layer 1, Layer 2, and Layer 3, showing a much more extensive area of damage than the other layers of the laminate. It was mainly attributed to the bending deformation of the laminate after being impacted, which made the bottom plate of the laminate satisfy the tensile failure at first and gradually expand to the upper plate with the increase in the impact time. It was noteworthy that the extended morphology of the matrix damage in each layer was consistent with the fiber layup direction of that layout. For example, the matrix tensile damage in Layer 1 extended along the 45-degree angle, and the matrix tensile damage in Layer 2 extended along the 0-degree angle.

#### 4.2.3. Interlaminar Damage

**Figure 12:**
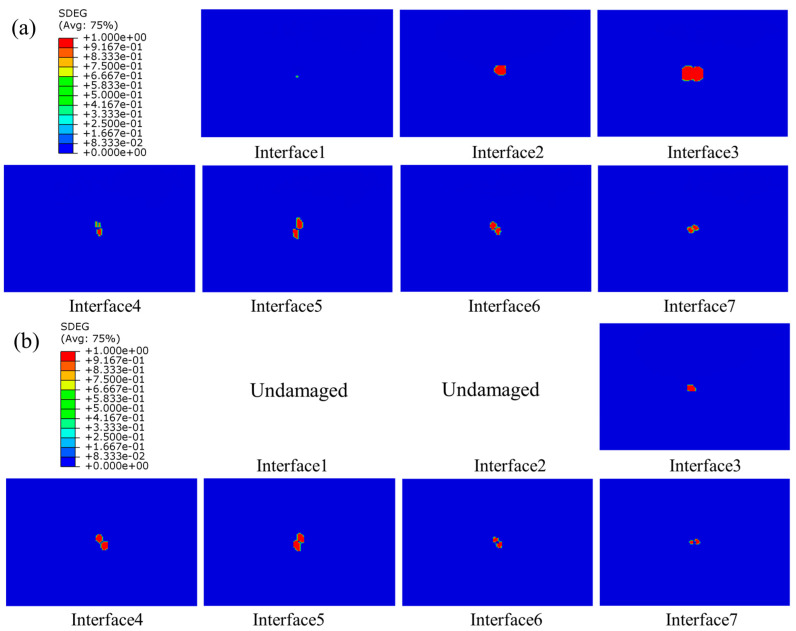
Delamination damage in stitched and unstitched composite laminates under 10 J. (**a**) Delamination damage in the group UL; (**b**) delamination damage in the group SL1010.

**Figure 13:**
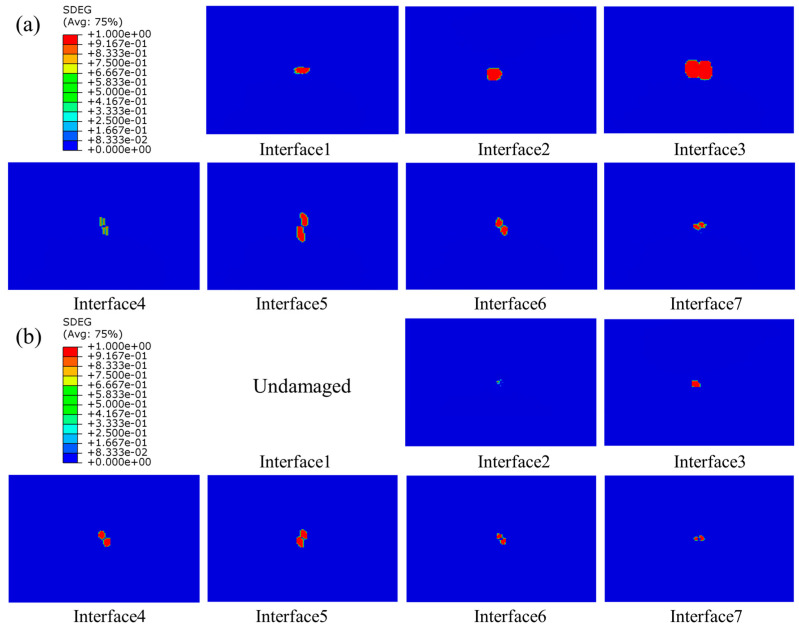
Delamination damage in stitched and unstitched composite laminates under 15 J. (**a**) Delamination damage in the group UL; (**b**) delamination damage in the group SL1010.

**Figure 14:**
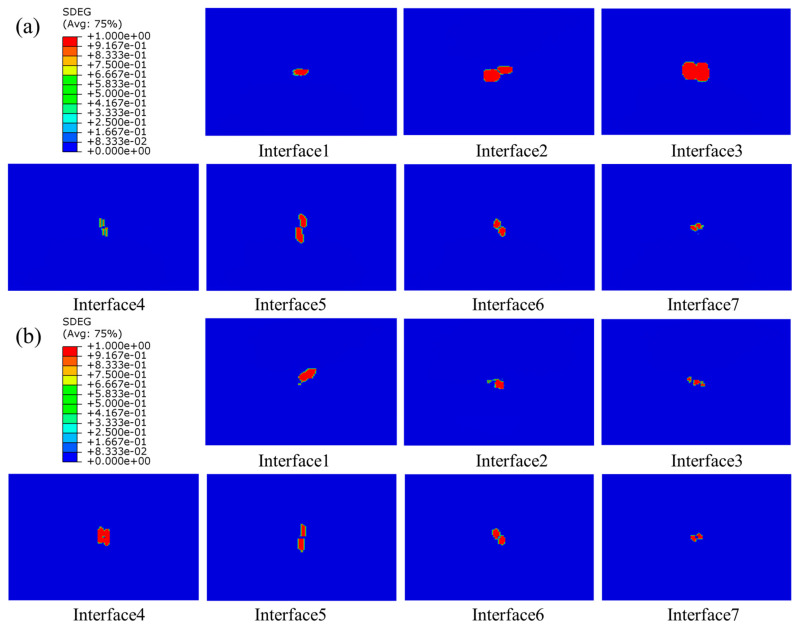
Delamination damage in stitched and unstitched composite laminates under 20 J. (**a**) Delamination damage in the group UL; (**b**) delamination damage in the group SL1010.

The delamination of composite laminates at three different energies of 10 J, 15 J, and 20 J are shown in Figure 12, Figure 13 and Figure 14, respectively. As seen in the figures, delamination damage existed in each layer of the unstitched group UL, with the delamination spreading from the center to the surroundings, displaying an irregularly shaped distribution. The largest delamination damage was in the second and third layers, and the area of delamination damage away from the impact surface was larger than those near to the top surface. The delamination damage morphology in the region close to the top surface was consistent with the direction of fibers in the upper plate of the adjacent layer. For example, the long-axis direction of the damage shape of Interface 5 was 90 degrees, which was consistent with the fiber direction of Layer 5, and the long-axis direction of the damage shape of Interface 6 was −45 degrees, which was also consistent with the fiber direction of Layer 6. No damage was produced on Interface 1 and 2 away from the impact surface in the SL1010 group under 10 J, and no damage was produced on Interface 1 away from the impact surface under 15 J. From the delamination damage of the stitched composites in three groups with different impact energies, it was evident that the stitches were able to effectively inhibit the delamination damage and reduce the area of delamination damage, especially in the three layers near the bottom surface.

### 4.3. Effect of Thicknesses on Mechanical Response and Damage of Composite Laminates

#### 4.3.1. Mechanical Response

**Figure 15:**
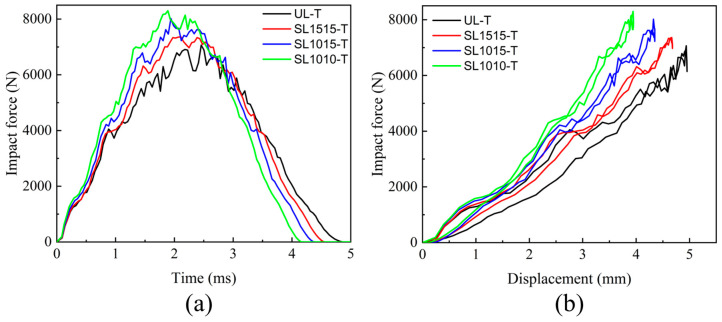
Mechanical response to the out-of-plane central impact under 20 J. (**a**) Impact force-time of unstitched and stitched composite laminates; (**b**) impact force-displacement of unstitched and stitched composite laminates.

The mechanical response of stitched and unstitched composite laminates under out-of-plane central impact at 20 J is presented in Figure 15, and the thickness of the laminates was 4.8 mm. By comparing Figure 15a with Figure 7e, it can be seen that the thicker the thickness of the laminate, the greater the peak impact force and the shorter the time to reach the peak impact force. The maximum displacement of SL1010-T was also smaller than that of SL1010, as can be seen from Figure 15b, indicating that the thicker the laminate was, the smaller the maximum displacement of the impactor was. This was mainly because the thick plate has a larger bending stiffness, the bending when subjected to impact was minor, and the deformation of the laminate mainly showed localized bending during the whole impact process. On the other hand, because of the thin thickness of the thin plate, the bending stiffness was smaller, and the bending deformation was larger when subjected to the impact, which resulted in a relatively large maximum displacement.

#### 4.3.2. Intralaminar Damage

**Figure 16:**
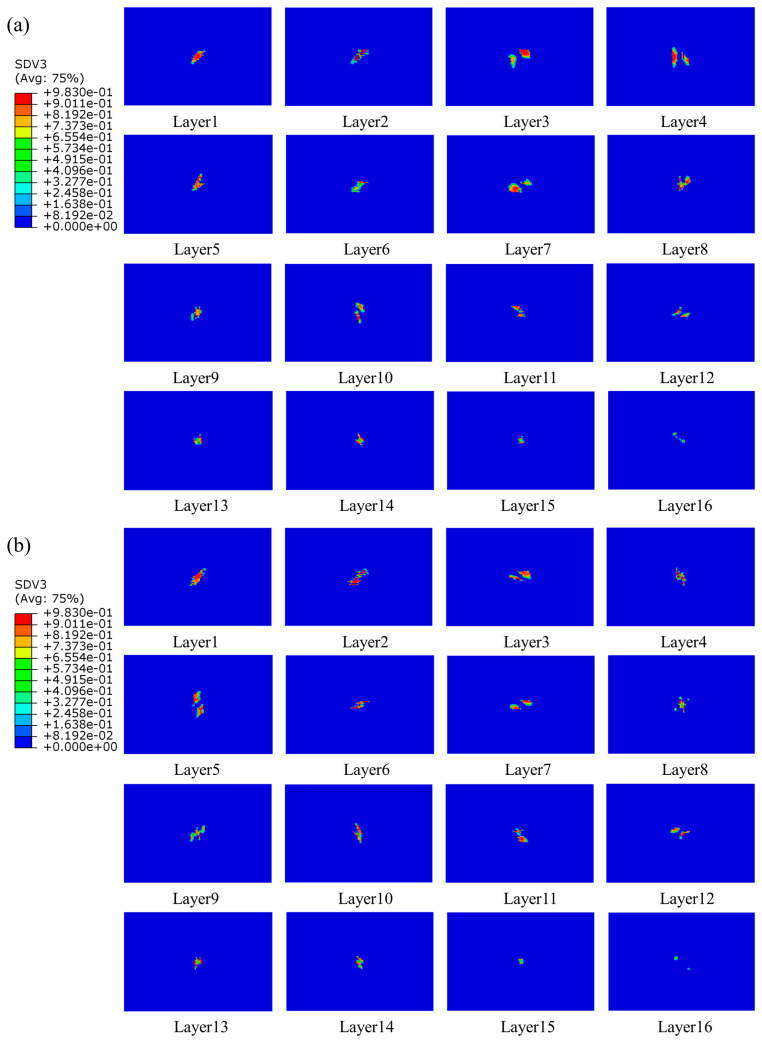
Matrix tensile damage of stitched and unstitched composite laminates with a thickness of 4.8 mm under 20 J. (**a**) Matrix tensile damage in group UL; (**b**) matrix tensile damage in group SL1010.

The damage to the composite laminates with a thickness of 4.8 mm was mainly characterized by matrix tensile damage, and no other forms of damage were revealed; the matrix tensile damage is displayed in Figure 16. Comparison with Figure 11 revealed that the main damage contours varied considerably with thickness, especially in the region near the bottom layer. It was mainly because of the bending deformation of the laminate during the impact process. The layer near the roof was unsusceptible for tensile damage, while the bottom layer was susceptible to tensile damage because of the large bending deformation. With the increase in thickness, the damage area was gradually reduced, and the damage close to the roof area gradually disappeared, which was because with the increased thickness, the thicker laminate had better impact resistance, and the matrix damage was also reduced. The matrix damage mode of composite laminates under the effect of low-speed impact was affected by the thickness, and the thinner the thickness of the laminate, the more susceptible to matrix damage.

#### 4.3.3. Interlaminar Damage

**Figure 17:**
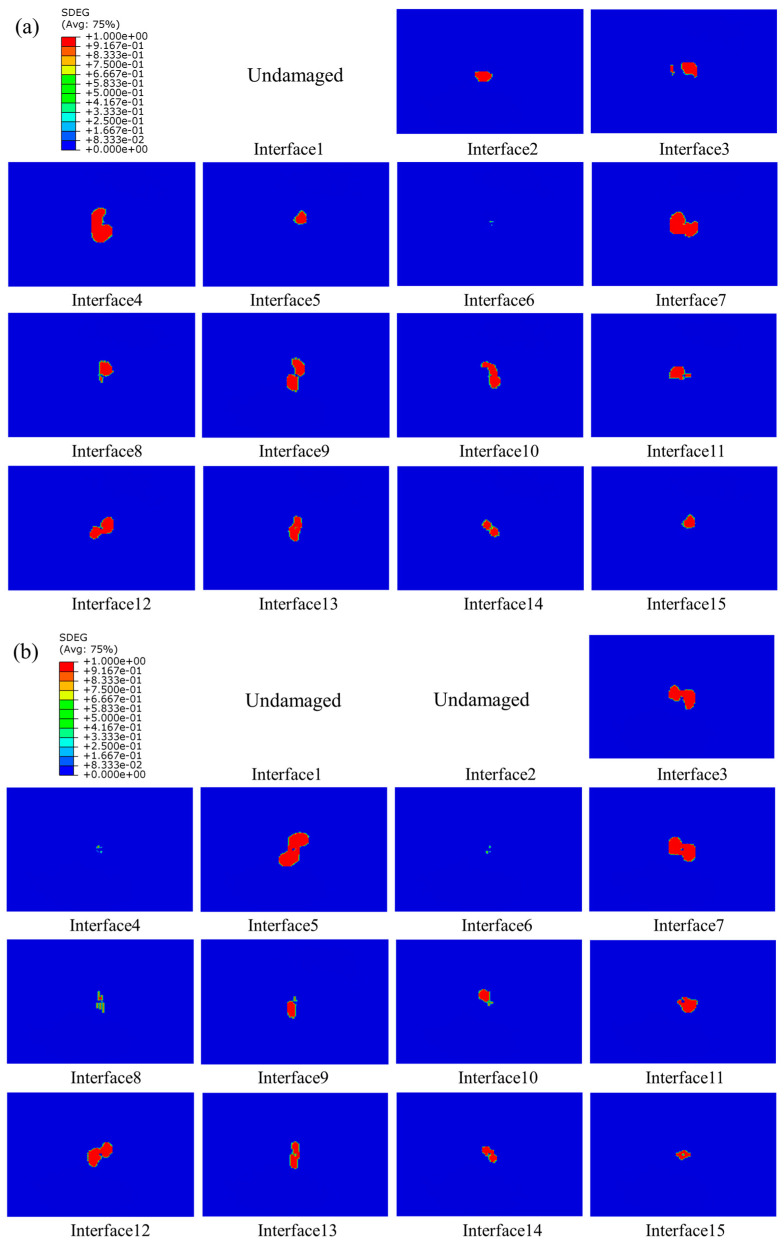
Delamination damage of stitched and unstitched composite laminates with a thickness of 4.8 mm under 20 J. (**a**) Delamination damage in group UL; (**b**) delamination damage in group SL1010.

The distribution of interlaminar damage in stitched and unstitched composite laminates with a thickness of 4.8 mm is represented in Figure 17. Comparison with Figure 14 revealed that the delamination damage morphology exhibited by laminates with different thicknesses varied. For thick plates, the delamination damage area was larger, and the long axis of the delamination damage geometry of the laminate near the top surface was in the direction of the fibers of the plate on the adjacent layer, a phenomenon that has also been reported in the literature [54]. This was mainly because the thicker the laminate, the greater the tensile stress on the laminate, leading to a wider stress space between the upper and lower neighboring layers, and the larger the damaged area. Therefore, under the same impact energy, the thin plate mainly depleted the impact energy through matrix damage, and the thick plate mainly consumed the impact energy through delamination damage.

## 5. Conclusions

In this work, the effect of stitch density and thickness on the out-of-face center impact damage of stitched carbon fiber composite laminates is investigated using simulations, and the following conclusions are drawn:

(1) The finite element analysis model of an out-of-plane central impact applicable to carbon fiber stitched composite laminates was developed. The VUMAT subroutine was coded, and the subroutine adopted the 3D Hashin and Hou failure criterion as the initiation damage criterion, combined with the linear degradation scheme of equivalent displacement for stiffness degradation and the cohesive zone model to simulate the interlaminar condition. Comparing the experimental results, the predicted mechanical responses obtained with the model matched well, which verified the correctness of the model.
(2) The internal damage of the laminate became more severe with the increased impact energy. The stitching process was beneficial in improving the impact tolerance of the laminate and inhibiting the delamination damage. In addition, the improvement effect was more obvious with the increased impact energy. The stitched composites could bear a higher peak force at the same energy, and it increased with the increase in the stitching density.
(3) The internal damage of stitched composite laminates was mainly manifested as matrix tensile damage. The damage of the bottom layer was more than that of the top layer when the laminate suffered from out-of-face central impact, mainly because the laminate was subjected to bending, which made the bottom layer of the laminate satisfy the tensile fail first, and gradually expand to the top layer with the increasing of impact time.
(4) The bending stiffness of the thin plate was less, and the matrix tensile damage was relatively significant; the bending stiffness of the thick plate was greater, and the delamination damage was relatively extensive. The energy of the impact was dissipated through the delamination.

## Figures and Tables

**Table 1:** Material parameters of CF1200-6300/R668 unidirectional [22].

**Unidirectional Lamia**	
Density (kg·m^−3^)	1760
Young’s modulus (GPa)	*E*_11_ = 123; *E*_22_ = *E*_33_ = 10.1; *G*_12_ = *G*_13_ = 4.6; *G*_23_ = 3.082
Poisson’s ratio	*μ*_12_ = *μ*_13_ = 0.28; *μ*_23_ = 0.21;
Strength (GPa)	*X*_T_ = 2260; *X*_C_ = 1370; *Y*_T_ = 51; *Y*_C_ = 130; *S*_12_ = 68; *S*_13_ = S_23_ = 40
**Interface**	
Young’s modulus (GPa)	*E* = 9.5; *G* = 8.1
Strength (GPa)	*N* = 50; *S* = 110
Fracture energy (N·mm^−1^)	$G_{n}^{c}=0.27;\;G_{s}^{c}=0.49$

**Table 2:** Material parameters of stitching resin cylinder.

Material Parameters	Kevlar29	R688-H3268	Equivalent Tricot Resin Cylinder
Young’s modulus (GPa)	70.50	3.90	50.52
Strength (GPa)	2.92	0.08	2.07
Poisson’s ratio	0.36	0.30	0.34
Density (kg·m^−3^)	1440	1065	1328

## Data Availability

Data are contained within the article.
